# Maternity waiting homes as part of a comprehensive approach to maternal and newborn care: a cross-sectional survey

**DOI:** 10.1186/s12884-019-2384-6

**Published:** 2019-07-04

**Authors:** Jody R. Lori, Joseph Perosky, Michelle L. Munro-Kramer, Phil Veliz, Gertrude Musonda, Jameson Kaunda, Carol J. Boyd, Rachael Bonawitz, Godfrey Biemba, Thandiwe Ngoma, Nancy Scott

**Affiliations:** 1 Office of Global Affairs, PAHO/WHO Collaborating Center, School of Nursing, 400 N. Ingalls, University of Michigan, Ann Arbor, MI 48109 USA; 2 School of Nursing, University of Michigan, 400 N. Ingalls, Ann Arbor, MI 48109 USA; 3 Africare-Zambia, Flat A, Plot 2407/10 MBX, Off Twin Palm Road, Ibex Hill, Box, 33921 Lusaka, Zambia; 4 Center for the Study of Drugs, Alcohol, Smoking and Health, School of Nursing, University of Michigan, Ann Arbor, MI 48109 USA; 5 Institute for Research on Women and Gender, University of Michigan, Ann Arbor, MI 48109 USA; 6 School of Public Health, Boston University, Boston, MA 02118 USA; 7 Boston University, School of Public Health, Director/CEO, National Health Research Authority (NHRA), Lusaka, Zambia; 8 Right to Care Zambia, Lusaka, Zambia

**Keywords:** Antenatal care, Maternity waiting homes, Postnatal care, Zambia

## Abstract

**Background:**

Increased encounters with the healthcare system at multiple levels have the potential to improve maternal and newborn outcomes. The literature is replete with evidence on the impact of antenatal care and postnatal care to improve outcomes. Additionally, maternity waiting homes (MWHs) have been identified as a critical link in the continuum of care for maternal and newborn health yet there is scant data on the associations among MWH use and antenatal/postnatal attendance, family planning and immunization rates of newborns.

**Methods:**

A cross-sectional household survey was conducted to collect data from women who delivered a child in the past 13 months from catchment areas associated with 40 healthcare facilities in seven rural Saving Mothers Giving Life districts in Zambia. Multi-stage random sampling procedures were employed with a final sample of *n* = 2381. Logistic regression models with adjusted odds ratios and 95% confidence intervals were used to analyze the data.

**Results:**

The use of a MWH was associated with increased odds of attending four or more antenatal care visits (OR = 1.45, 95% CI = 1.26, 1.68), attending all postnatal care check-ups (OR = 2.00, 95% CI = 1.29, 3.12) and taking measures to avoid pregnancy (OR = 1.31, 95% CI = 1.10, 1.55) when compared to participants who did not use a MWH.

**Conclusions:**

This is the first study to quantitatively examine the relationship between the use of MWHs and antenatal and postnatal uptake. Developing a comprehensive package of services for maternal and newborn care has the potential to improve acceptability, accessibility, and availability of healthcare services for maternal and newborn health. Maternity waiting homes have the potential to be used as part of a multi-pronged approach to improve maternal and newborn outcomes.

**Trial registration:**

National Institutes of Health Trial Registration NCT02620436, Impact Evaluation of Maternity Homes Access in Zambia, Date of Registration - December 3, 2015.

## Background

It is well recognized that underutilization of life-saving health services has been associated with poor maternal and newborn outcomes [1]: however, multiple factors influence the use of maternal health services for women living in poor, remote communities. Zambia is a country with underutilization of health services; according to the Partnership for Maternal, Newborn and Child Health, Zambia is one of 49 global strategy priority countries [2]. The maternal mortality ratio in Zambia is 224 per 100,000 live births and infant mortality is reported at 45 deaths per 1000 live births [3]. The number of births projected for 2015 was 656,428 [4].

It has been established that antenatal care (ANC) can save lives by implementing timely and appropriate evidence-based practices [5]. Recently in Zambia, coverage of ANC significantly increased through efforts of the Millennium Development Goals (MDGs), with 96% of mothers attending at least one ANC visit with a skilled provider in 2015 [6]. However, according to the most recent Demographic and Health Survey only 55.5% of women attended four or more ANC visits [7], the number of visits deemed the most beneficial by the World Health Organization (WHO) until recently [5].

In addition to the importance of ANC care, the critical importance of the postnatal period is well established, with prior evidence suggesting that 60% of global maternal deaths occur during the postnatal period [8]. WHO recommends four postnatal checkups within the first six weeks on the following schedule: day 1 (24 h), day 3 (48–72 h), between days 7–14 and at six weeks postpartum [9]. An integral part of this postnatal care (PNC) is the provision of contraceptive education to postpartum women and routine vaccinations for their newborns [10]. In Zambia, 63% of women receive PNC in the critical first two days after delivery. Of these, the majority (48%) are seen in the first four hours following delivery, 14% receive care within 4–23 h, and 2% are seen 1–2 days following delivery [7].

Maternity waiting homes (MWHs), also known as mother’s shelters, are structures built near healthcare facilities to minimize the critical barrier of distance to accessing maternal health services. They serve as one potential health intervention that may be incorporated into a package of maternal and newborn health services. The Zambian government has identified MWHs as an intervention to increase demand for maternity care services, improve geographic access to facility delivery, and address the second delay: delay in reaching a health facility, first identified by Thaddeus and Maine in the three-delay model [11].

While WHO recognizes the value of MWHs as a critical link in the continuum of care, bringing women closer to healthcare facilities near the time of delivery [12], MWHs can contribute to a larger health system strengthening effort to connect women to the health facility and to ANC and PNC services for both mothers and newborns. However, to date there is a dearth of literature documenting the relationship between the use of MWHs and ANC and PNC utilization. In theory, MWHs may be an important link within the continuum of care; however, there is scant data on whether women engage in all three services. Therefore, the objective of this study was to assess the associations among MWH use and ANC and PNC attendance, family planning, and immunization rates of newborns for mothers living in seven rural districts in Zambia.

## Methods

A cross-sectional household survey design was used to collect data from women who delivered a child in the past 13 months from catchment areas associated with 40 healthcare facilities in seven rural districts that are part of the Saving Mothers, Giving Life (SMGL) initiative in Zambia: Choma, Kalomo, Lundazi, Mansa, Nyimba, Pemba, and Chembe in three provinces (Eastern, Luapula, and Southern) [13]. The SMGL initiative, launched in 2012, takes a health systems approach to improve access to safe, clean childbirth and timely emergency care. SMGL district study sites were selected for the study through formative reseach conducted in 2013–2014 [14, 15]. The research methodology is described in detail elsewhere [16, 17]. Ethical approval was obtained from Boston University Institutional Review Board (IRB), University of Michigan IRB, and the ERES Converge IRB in Zambia.

### Study sample and setting

Multi-stage random sampling procedures were employed with probability proportionate to population size. First, the sampling frame of villages within the health facility catchment area located more than 9.5 km from the health facility, was derived through geo-coding; approximately 10 village clusters were randomly selected from each catchment area. In the second stage of sampling, all potentially eligible households within the selected villages were listed, randomly ordered, and then approached to contact an eligible participant. If more than one eligible participant was in the household, a single participant was randomly sampled. Inclusion criteria consisted of: 1) delivered within the past 13 months regardless of maternal or neonatal outcome OR a proxy participant if the woman was deceased (regardless of maternal or newborn outcome); 2) 15 years of age or older; if age 15–17, a legal guardian available to consent (proxy participants 18 years or older; 3) resident of a village 9.5 km or farther from the catchment area health facility. All eligible participants provided written informed consent prior to any survey procedures. Each survey took approximately 45 min. Participants received a small token of appreciation, equivalent to $2 USD in acknowledgment of their time.

### Data collection

A team of local research assistants, literate in the appropriate local languages and English, were trained in human subjects’ protection and data collection methods during a 5-day training. Data were captured electronically using SurveyCTO Collect Software installed on encrypted tablets in early 2016.

### Measures

#### Dependent variables – antenatal and postnatal care

Several measures were used to assess the participants’ use of ANC or PNC during their most recent pregnancy (within the past 13 months). Below are the items used to construct the dependent variables used in this analysis.

#### Frequency of antenatal care

A question asked participants the number of times they attended ANC at a health facility or health post. The response options included “none”, “one time”, “two times”, “three times”, “four times”, and “more than four times”. To capture the frequency of ANC visits for women, we dichotomized responses into those who attended ANC four or more times versus those who attended ANC three times or less.

#### Postnatal care visits

Participants could respond ‘yes’ or ‘no’ to questions regarding whether they went to a health facility or health post for “ANY postnatal checks after the first 24 hours following your last delivery”; “a postnatal check approximately 3 days after your last delivery”; “a postnatal check between 7 and 14 days after your last delivery”; and “a postnatal check approximately 6 weeks after you last delivery”, aligned with WHO guidelines [9]. Two variables were created: one to assess any postnatal checks (yes versus no) and one to indicate attending all postnatal checks (visits 24 h after delivery, 3 days, 7 to 14 days, and 6 weeks after their last delivery).

#### Contraception/avoiding pregnancy

Participants were asked if they “currently use something or try in any way to delay or avoid getting pregnant?” The response options included “yes” and “no”. This measure was treated as a dichotomous variable in the analyses.

#### Vaccinations for child

An additional question asked participants if their “… child received any vaccinations?” Response options were binary and included “yes” and “no” to assess if participants indicated that their child received specific vaccinations at birth (i.e., BCG and OPV-0), 6 weeks (i.e., OPV-1, DTP-HepB-Hib-1, PCV, and Rotavirus), 10 weeks (i.e., OPV-2, DTP-HepB-Hib-2, PCV, and Rotavirus), and 14 weeks (i.e., OPV-3, DTP-HepB-Hib-3, and PCV), a binary variable (yes and no) was constructed to distinguish whether participants indicated receiving all of the required vaccinations for their child depending on age versus those who did not complete the full cycle of vaccinations for their child. Responses were checked against vaccination cards in 79% of cases (*n* = 1677) and controlled for infant status at time of survey. Mothers whose babies died (*n* = 48) were excluded from the immunization analysis.

#### Independent variables – maternity waiting home use

The primary independent variable was whether the mother used a MWH for her most recent delivery. Participants were asked if they stayed “at a mothers’ shelter for any reason at all before or after that delivery.” Participants had two response options: “yes” and “no”. The variable was treated as a dichotomous variable in the analyses outlined below.

### Control variables

Control variables included household size, marital status, number of births, sex of the head of household, age, and educational level for the mothers who participated in the study.

### Data analysis

STATA 15.0 was used to estimate the models outlined above (Version 15.0; StataCorp LP, College Station, Texas). All logistic regression models included adjusted odds ratios (AOR) and 95% confidence intervals (95% CI). Additionally, models accounted for clustering within each of the seven districts where the sample of participants were obtained.

## Results

The response rate was 86.9% with a final sample of 2381 participants from unique households. Of those eligible but who did not respond: 280 (10.2%) were unavailable primarily due to their work in the fields for the harvest, 60 (2.2%) refused participation, and 20 (0.7%) withdrew after beginning the survey or had incomplete surveys and were dropped from the analysis.

Overall, 58.6% of our sample attended four or more ANC visits; 3.6% of participants attended ANC between 0 and 1 time, while 37.8% attended ANC between 2 and 3 times. Over 45% reported at least one PNC visit (14.2% attended 1 to 2 PNC visits, 25.7% attended three PNC visits, and 6.4% went to all PNC visits) with respect to their most recent pregnancy and birth (Table 1). Approximately 34.5% of participants indicated using some form of contraception after their most recent birth. The majority (91.1%) reported immunizing their newborn; however, among the sample of participants whose child was 14 weeks of age or older, only 39.1% indicated their child had received all of the required vaccinations. Participants reported 65.2% of newborns received all recommended vaccinations at birth (BCG and Oral Polio [OPV-0]); 83.6% received the full recommended list at 6-weeks (OPV-1, DPT-HepB-Hib-1); 73.8% at 10-weeks (OPV-2, DPT-HepB-Hib2); and 57.4% at 14-weeks (OPV-3, DPT-HepB-Hib3).

**Table 1:** Bivariate analysis with socio-demographic variables

*N* = 2381	Attended four or more ANC visits	Attended ***ANY*** PNC Visit	Attended ***ALL*** PNC Visits	Avoiding Pregnancy	Child received ***ANY*** vaccinations	Child received ***ALL*** vaccinations^1^
	Percent (95% CI)	Percent (95% CI)	Percent (95% CI)	Percent (95% CI)	Percent (95% CI)	Percent (95% CI)
Total	58.6% (55.1–62.1)	45.3% (35.3–55.8)	6.4% (3.4–11.6)	34.5% (25.9–44.3)	91.1% (86.6–94.2)	39.1% (28.9–50.3)
	% OR (95% CI)	% OR (95% CI)	% OR (95% CI)	% OR (95% CI)	% OR (95% CI)	% OR (95% CI)
Household Size						
1 to 3 people (11.5%)	64.9% Reference	51.1% Reference	9.3% Reference	40.8% Reference	91.9% Reference	41.7% Reference
4 to 6 people (39.5%)	57.3% .723* (.536, .975)	46.1% .820 (.665, 1.01)	6.3% .654 (.421, 1.01)	37.7% .876 (.665, 1.15)	91.4% .942 (.544, 1.62)	37.2% .829 (.453, 1.52)
7 or more people (49.0%)	58.2% .751 (.497, 1.13)	43.3% .730** (.585, .911)	5.8% .600 (.318, 1.13)	30.3% .630** (.445, .891)	90.7% .857 (.558, 1.31)	40.0% .934 (.651, 1.34)
Marital Status						
Married (88.0%)	59.1% Reference	45.5% Reference	6.6% Reference	37.1% Reference	91.4% Reference	38.5% Reference
Not Married (12.0%)	54.8% .838 (.614, 1.14)	44.0% .940 (.675, 1.30)	5.1% .758 (.404, 1.42)	14.7% .293*** (.208, .411)	88.8% .749 (.450, 1.24)	43.4% 1.21 (.948, 1.56)
Number of Births						
At least 1 (23.2%)	60.5% Reference	50.0% Reference	6.9% Reference	29.7% Reference	89.3% Reference	41.2% Reference
2 or 3 (31.8%)	57.2% .872 (.748, 1.01)	44.0% .783* (.633, .969)	5.4% .766 (.464, 1.26)	39.4% 1.54*** (1.35, 1.76)	92.2% 1.40*** (1.15, 1.70)	40.7% .979 (.700, 1.36)
4 or more (45.0%)	58.5% .922 (.670, 1.26)	44.0% .783** (.656, .934)	6.8% .992 (.697, 1.41)	33.4% 1.18* (1.01, 1.38)	91.3% 1.25* (1.04, 1.50)	37.2% .847 (.639, 1.12)
Head of Household						
Male (76.6%)	58.8% Reference	43.8% Reference	5.8% Reference	37.4% Reference	91.6% Reference	38.6% Reference
Female (9.7%)	58.1% .967 (.693, 1.35)	57.6% 1.73***(1.45, 2.08)	14.8% 2.83***(1.51,5.29)	32.7% .812 (.568, 1.16)	92.1% 1.07 (.759, 1.51)	41.7% 1.13 (.724, 1.79)
Unknown (13.6%)	57.7% .953 (.671, 1.35)	44.6% 1.02 (.806, 1.31)	3.5% .586 (.328,1.04)	18.8% .388*** (.283, .532)	87.7% .657 (.378, 1.13)	40.1% 1.06 (.768, 1.47)
Age						
15 to 19 (17.9%)	57.4% Reference	46.5% Reference	5.5% Reference	27.5% Reference	89.6% Reference	35.8% Reference
20 to 24 (32.1%)	59.2% 1.07 (.814, 1.41)	46.2% .987 (.705, 1.38)	6.7% 1.21 (.609, 2.42)	39.4% 1.71*** (1.39, 2.10)	90.4% 1.09 (.841, 1.42)	39.4% 1.16 (.893, 1.51)
25 to 29 (19.1%)	56.8% .976 (.821, 1.15)	45.4% .960 (.864, 1.06)	5.6% 1.01 (.546, 1.89)	34.3% 1.37*** (1.20, 1.56)	91.6% 1.26** (1.06, 1.50)	42.7% 1.33 (.913, 1.95)
30 to 34 (16.1%)	58.9% 1.06 (.745, 1.51)	46.6% 1.00 (.711, 1.42)	6.9% 1.26 (.582, 2.73)	34.9% 1.40*** (1.18, 1.66)	93.6% 1.69* (1.03, 2.77)	33.8% .915 (.621, 1.34)
35 and older (14.7%)	60.1% 1.11 (.849, 1.47)	40.4% .781 (.544, 1.12)	6.9% 1.27 (.577, 2.79)	31.2% 1.19 (.903, 1.57)	90.7% 1.13 (.777, 1.65)	43.4% 1.37 (.868, 2.17)
Education						
No Education (15.2%)	54.9% Reference	44.9% Reference	7.8% Reference	30.1% Reference	89.6% Reference	39.9% Reference
Some Primary (40.8%)	57.5% 1.10 (.885, 1.38)	47.0% 1.08 (.792, 1.49)	6.4% .805 (.573, 1.12)	36.1% 1.31*** (1.17, 1.47)	92.7% 1.47* (1.08, 1.99)	38.5% .943 (.768, 1.15)
Completed Primary (44.0%)	60.9% 1.27 (1.01, 1.60)	43.9% .958 (.704, 1.30)	5.8% .722 (.506, 1.02)	34.4% 1.22 (.990, 1.50)	90.2% 1.07 (.568, 2.01)	39.3% .977 (.708, 1.34)

*p < .05, **p < .01, ***p < .001; All analyses use robust cluster to account for potential differences across households sampled across the seven districts

^1^Analyses assessing whether a child received all required vaccinations only used the sample of mothers whose children where 14 weeks old or older. 67.1% of the mothers’ most recent births included children

14 weeks or older (*n* = 1592)

Assessing the bivariate associations between sociodemographic characteristics and ANC and PNC reveals several statistically significant associations. Larger household size was negatively associated with attending four or more ANC visits, attending any PNC visit, and using some form of contraception to avoid pregnancy (Table 1). Participants living in households with seven or more people had lower odds of attending any PNC visit (OR = .730, 95% CI = .585, .911) and of attempting to prevent pregnancy (OR = .630, 95% CI = .445, .891) when compared to women living in smaller households of 1–3 people. Marital status was only associated with attempting to prevent pregnancy; mothers who were not married had lower odds of attempting to prevent pregnancy when compared to mothers who were married (OR = .293, 95% CI = .208, .411).

Number of previous births was associated with several of the listed outcomes in Table 1. Participants with two or more children were less likely to attend any PNC visit (2–3 children, OR = .783, 95% CI = .633, .969; 4 or more children, OR = .783, 95% CI = .656, .934) but significantly more likely to use contraception (2–3 children, OR = .1.54, 95% CI = 1.35, 1.76; 4 or more children, OR = 1.18, 95% CI = 1.01, 1.38), and to have their child immunized (2–3 children, OR = 1.40, 95% CI = 1.15, 1.70, 4 or more children, OR = 1.25, 95% CI = 1.04, 1.50) than women with only one child.

Participants who lived in households headed by females had higher odds of attending any PNC visit (OR = 1.73, 95% CI = 1.45, 2.08) and attending all four PNC check-ups (OR = 2.83, 95% CI = 1.51, 5.29), while those who lived in households where a head of household could not be identified (response reported as ‘unknown’) had lower odds of taking measures to avoid pregnancy when compared to mothers who lived in households headed by males (OR = .388, 95% CI = .283, .532).

Maternal age had a significant influence on attempting to avoid pregnancy with participants aged 20–30 years all having higher odds of avoiding pregnancy when compared to those aged 15–19 (20–24 years, OR = 1.71, 95% CI = 1.39, 2.10; 25–29 years, OR = 1.37, 95% CI = 1.20, 1.56; 30–34 years, OR = 1.40, 95% CI = 1.18, 1.66). Age also had a significant influence on childhood immunizations with participants 25–29 years and 30–34 years reporting higher levels of any childhood immunizations (OR = 1.26, 95% CI = 1.06, 1.50 and OR = 1.69, 95% CI = 1.03, 2.77 respectively).

Finally, participants’ level of education was positively associated with avoiding pregnancy and reporting any immunizations for their child. In particular, participants who had some primary education had higher odds of avoiding pregnancy (OR = 1.31, 95% CI = 1.17, 1.47) and obtaining any childhood immunizations for their most recent birth (OR = 1.47, 95% CI = 1.08, 1.99) when compared to those with no education.

The associations between using a MWH during the most recent pregnancy and utilization of both ANC and PNC are included in Table 2 . Among participants in the sample, 31.5% indicated using a MWH for their most recent pregnancy. The results indicate the use of a MWH was associated with increased odds of attending four or more ANC visits (OR = 1.45, 95% CI = 1.26, 1.68), attending all PNC check-ups (OR = 2.00, 95% CI = 1.29, 3.12), and taking measures to avoid pregnancy (OR = 1.31, 95% CI = 1.10, 1.55) when compared to participants who did not use a MWH.

**Table 2:** Bivariate analysis assessing how MWH is associated with PNC/ANC outcomes

N = 2381	Attended four or more ANC visits	Attended ***ANY*** PNC Visit	Attended ***ALL*** PNC Visits	Avoiding Pregnancy	Child received ***ANY*** vaccinations	Child received ***ALL*** vaccinations^1^
	Percent (95% CI)	Percent (95% CI)	Percent (95% CI)	Percent (95% CI)	Percent (95% CI)	Percent (95% CI)
Total	58.6% (55.1–62.1)	45.3% (35.3–55.8)	6.4% (3.4–11.6)	34.5% (25.9–44.3)	91.1% (86.6–94.2)	39.1% (28.9–50.3)
	Binary Logistic Regression	Binary Logistic Regression	Binary Logistic Regression	Binary Logistic Regression	Binary Logistic Regression	Binary Logistic Regression
	% OR (95% CI)	% OR (95% CI)	% OR (95% CI)	% OR (95% CI)	% OR (95% CI)	% OR (95% CI)
Used a MWH						
No (68.5%)	55.8% Reference	44.3% Reference	4.9% Reference	32.5% Reference	90.4% Reference	38.1% Reference
Yes (31.5%)	64.8% 1.45*** (1.26, 1.68)	47.4% 1.13 (.796, 1.61)	9.4% 2.00** (1.29, 3.12)	38.7% 1.31** (1.10, 1.55)	92.6% 1.32 (.977, 1.80)	41.4% 1.14 (.946, 1.38)

*p < .05, **p < .01, ***p < .001; All analyses use robust cluster to account for potential differences across households sampled across the seven districts

^1^Analyses assessing whether a child received all required vaccinations only used the sample of mothers whose children where 14 weeks old or older. 67.1% of the mothers’ most recent births included children

14 weeks or older (*n* = 1592)

After controlling for household size, marital status, number of births, sex of the head of household, maternal age, and educational level, the observed associations between MWH use and the increased odds of attending four or more ANC visits (AOR = 1.43, 95% CI = 1.25, 1.65), attending all PNC check-ups (AOR = 1.99, 95% CI = 1.30, 3.07), and taking measures to avoid pregnancy (AOR = 1.27, 95% CI = 1.08, 1.50) remained significant. Moreover, several sociodemographic variables still predict several of the ANC and PNC outcomes. Young mothers, between the ages of 15 and 19, had lower odds of optimum utilization of ANC and PNC when compared to participants who were in older age groups. Larger household size, unmarried status, and residing in households headed by males also show significantly lower odds related to the postnatal outcomes (Table 3).

**Table 3:** Multiple logistic regression assessing the association between MWH use and PNC/ANC outcomes

	Attended four or more ANC visits		Attended ***ANY*** PNC Visit		Attended ***ALL*** PNC Visits		Avoiding Pregnancy		Child received ***ANY*** vaccinations		Child received ***ALL*** vaccinations^1^	
	AOR	(95% CI)	AOR	(95% CI)	AOR	(95% CI)	AOR	(95% CI)	AOR	(95% CI)	AOR	(95% CI)
	*n* = 2346		*n* = 2313		n = 2313		*n* = 2344		*n* = 2315		*n* = 1523^2^	
Used a MWH												
No	Reference		Reference		Reference		Reference		Reference		Reference	
Yes	1.43*** (1.25, 1.65)		1.12 (.785, 1.61)		1.99** (1.30, 3.07)		1.27** (1.08, 1.50)		1.28 (.970, 1.71)		1.17 (.949, 1.45)	
Household Size												
1 to 3 people	Reference		Reference		Reference		Reference		Reference		Reference	
4 to 6 people	.727 (.503, 1.05)		.929 (.706, 1.22)		.685 (.414, 1.13)		.730*** (.607, .878)		.702 (.482, 1.02)		.832 (.503, 1.37)	
7 or more people	.695 (.454, 1.06)		.841 (.550, 1.28)		.611 (.280, 1.33)		.647*** (.503, .831)		.656* (.445, .966)		1.00 (.934, 1.08)	
Marital Status												
Married	Reference		Reference		Reference		Reference		Reference		Reference	
Not Married	.741 (.487, 1.12)		.727 (.518, 1.02)		.785 (.402, 1.53)		.370*** (.275, .497)		1.15 (.809, 1.65)		1.36 (.977, 1.92)	
Number of Births												
At least 1	Reference		Reference		Reference		Reference		Reference		Reference	
2 or 3	.880 (.752, 1.02)		.697 (.481, 1.00)		.720 (.318, 1.62)		1.24 (.964, 1.60)		1.37 (.965, 1.96)		.802 (.539, 1.19)	
4 or more	.945 (.596, 1.49)		.698 (.471, 1.03)		.903 (.528, 1.54)		1.03 (.624, 1.71)		.902 (.571, 1.42)		.523** (.326, .840)	
Head of Household												
Male	Reference		Reference		Reference		Reference		Reference		Reference	
Female	1.00 (.695, 1.44)		1.91*** (1.58, 2.32)		3.26*** (1.64, 6.47)		1.00 (.676, 1.50)		.990 (.712, 1.37)		1.07 (.666, 1.72)	
Unknown	1.32 (.918, 1.91)		1.27 (.933, 1.73)		.876 (.396, 1.94)		.849 (.653, 1.10)		.751 (.352, 1.60)		.802 (.468, 1.37)	
Age												
15 to 19	Reference		Reference		Reference		Reference		Reference		Reference	
20 to 24	1.24* (1.00, 1.54)		1.21 (.791, 1.87)		1.52 (.638, 3.62)		1.40** (1.09, 1.78)		.957 (.705, 1.29)		1.44* (1.03, 2.01)	
25 to 29	1.19 (.903, 1.58)		1.29** (1.08, 1.55)		1.23 (.557, 2.74)		1.20 (.901, 1.61)		1.31 (.993, 1.75)		2.11** (1.20, 3.68)	
30 to 34	1.35* (1.05, 1.73)		1.35 (.915, 1.99)		1.39 (.527, 3.67)		1.38 (.962, 1.99)		1.95* (1.05, 3.63)		1.55 (.853, 2.82)	
35 and older	1.45* (1.05, 1.99)		1.06 (.814, 1.40)		1.40 (.572, 3.45)		1.17 (.811, 1.69)		1.32 (.551, 3.17)		2.32** (1.35, 3.96)	
Education												
No Education	Reference		Reference		Reference		Reference		Reference		Reference	
Some Primary	1.10 (.870, 1.39)		1.06 (.814, 1.39)		.759 (.513, 1.49)		1.28*** (1.10, 1.50)		1.44** (1.09, 1.90)		.945 (.780, 1.14)	
Completed Primary	1.29 (.979, 1.72)		.907 (.700, 1.17)		.721 (.704, 1.30)		1.27 (.905, 1.79)		1.08 (.600, 1.95)		.902 (.645, 1.26)	

*p < .05, **p < .01, ***p < .001; All analyses use robust cluster to account for potential differences across households sampled across the seven districts

^1^Analyses assessing whether a child received all required vaccinations only used the sample of mothers whose children where 14 weeks old or older. 67.1% of the mothers’ most recent births included children 14 weeks or older (n = 1592)

^2^Sample size is lower among the sample of mothers with children 14 weeks or older due to missing data on the covariates presented in Table 3

## Discussion

Thirty-one percent of our sample reported using a MWH during their last pregnancy. Results indicate a positive association between MWH use and number of ANC visits (four or more visits), attending all PNC visits, and increased contraceptive use of any kind to avoid pregnancy. Although directionality cannot be established, our results highlight the potential influence of a comprehensive package of services for women living in rural, remote areas.

Studies have shown a positive influence between attendance at ANC and PNC services and use of a skilled birth attendant for delivery as well as uptake of modern family planning methods [18, 19]. Additionally, the use of MWHs has been associated with an increase in facility delivery and skilled birth attendance [20, 21]. Maternity waiting homes as an intervention to increase facility-based delivery may provide a conduit for enhanced communication and community outreach, both of which have the potential to influence ANC, PNC, and family planning uptake [22].

Less than half (45.3%) of participants in our study attended any PNC, far less than the 63% of women reported in the Zambia Demographic and Health Survey [7]. This could be due to rugged terrain and long distances to healthcare facilities for participants living in the seven districts in our study. Just as geographic distance may decrease facility utilization for birth, that same distance may represent a challenge to attending the recommended number of PNC visits. Household size also had a significant effect on whether women attended PNC and their attempts to avoid pregnancy, with participants living in larger households less likely to attend PNC and to be using any form of contraception. One notable finding was that participants living in female-headed households were more likely than those from homes with a male head of household to attend postnatal care. This may be a reflection of women’s household position and their autonomy in decision-making [23].

Postnatal care is an effective intervention for childhood immunizations and the uptake of family planning services [18, 24]. In this study, the use of a MWH was associated with an increased uptake of PNC care and subsequently increased avoidance of pregnancy. Previous studies have reported an association between PNC care and modern contraceptive use in Kenya, Zambia, and Ethiopia [18, 24]. The findings of this study suggest the expansion of a comprehensive maternal, newborn, and child health package of service to include MWHs has the potential to further increase PNC care and use of contraceptive services.

Although there was an association between use of a MWH and attendance at all PNC visits, there was no relationship between MWH use and childhood immunizations. The immunization schedule in Zambia follows the WHO recommendations for childhood immunization [25]. Interestingly, 91% of subjects reported their infant received at least one childhood immunization. The lack of an increase in receiving at least one immunization with the use of a MWH may be due to a ceiling effect as well as the fact that the first immunizations including BCG and Hepatitis B are given immediately at birth while the mother and newborn are still at the health facility.

Developing a comprehensive package of services for maternal and newborn care has the potential to improve the availability, accessibility and acceptability of care for mothers and newborns in low-resource settings [26]. Increased encounters with the healthcare system at multiple levels have the potential to improve maternal and newborn outcomes.

## Limitations

This study has four main limitations. First, this is a cross-sectional study and we cannot assess directionality or change over time; however, it is unique in that it is a large sample size and contains numerous variables on a representative sample of a remote population. Second, this study is limited by its focus on the Saving Mothers Giving Life (SMGL) districts which constrains generalization beyond the SMGL districts selected for this study. The seven districts in our study are part of the 10 learning districts included in the SMGL five-year public private partnership to decrease maternal mortality by 50% and perinatal mortality by 30% in Uganda and Zambia with a plan to then scale up nationally in both countries [13]. These districts have had considerable resources provided over the course of the past five years and it is likely that outcomes in these districts are better than others. Additionally, several questions relied on participant’s recall of events over the past 13 months potentially affecting the accuracy or completeness of the recollections retrieved by participants as well as the possibility of social desirability bias. Finally, at the end of this study, there were no national policies regarding standardization of MWHs in Zambia, therefore wide variation could exist at each MWH.

## Conclusions

This is the first study to quantitatively examine the relationship between the use of MWHs and ANC and PNC utilization. Providing a comprehensive approach to maternal and newborn health with the appropriate utilization of services can have a positive impact on maternal and newborn outcomes [19]. Our findings suggest MWHs are associated with some healthy behaviors among women who use them (e.g., attendance at four or more ANC visits, attendance at all PNC visits, and taking measures to avoid pregnancy). Maternity waiting homes can be used as part of a multi-pronged approach to improving maternal and newborn services, ultimately increasing access to and attendance at ANC and PNC. By taking a holistic approach to maternal and newborn services, MWHs have the potential to increase contacts with the healthcare system and improve maternal and newborn outcomes. Future research is needed on the cost implications of such a strategy and the long term sustainability of MWHs for resource poor settings such as Zambia.

## Data Availability

Data used for this manuscript are part of an on-going clinical trial. The Boston University IRB and the ERES Converge IRB in Zambia approved that data would only be presented in aggregate form. Additionally, the consent forms explicitly state the data will be only shared in aggregate form. Once the clinical trial is complete and primary results have been disseminated the data will be released. Data requests may be sent to the Boston University IRB at: medirb@bu.edu. We would also like to confirm we will be able to share the minimal anonymized data set if and when our manuscript is accepted for publication.

## Abbreviations

ANC: Antenatal care
AOR: Adjusted odds ratio
BCG: Bacille Calmette-Guérin
CI: Confidence interval
DPT: Diphtheria, Pertussis, Tetanus
HepB: Hepatitis B
Hib: Haemophilus-influenzae-type-b
IRB: Institutional Review Board
MDG: Millennium Development Goals
MWH: Maternity waiting home
OPV: Oral polio vaccine
OR: Odds ratio
PNC: Postnatal care
SMGL: Saving Mothers, Giving Life
USD: United States dollar
WHO: World Health Organization
